# On the Treatment of Gleet

**Published:** 1855-04

**Authors:** 


On the Treatment of Gleet. By M. Ricord.—

[M. Calvo, a private pupil of M. Ricord, has recently published in the *Moniteur des Hopitaux* reports of the lectures at the *Hôpital du Midi* during 1854. Here are some useful points in regard to the management of blennorrhœa, which we take from the number for September 12th of the journal alluded to. On a future occasion we may make further extracts.]

Notwithstanding the most judicious treatment, blennorhagia often passes into the chronic stage, and becomes the intractable disorder termed blennorrhœa, *goutte militaire*, or gleet.

When this occurs, it is proper in the first place to examine the meatus of the urethra; the lips of this orifice should be separated, and if any inflamed follicles are perceived, they should be incised with a cataract needle and cauterized with nitrate of silver.

If the discharge continues, it will be necessary to institute an internal treatment. The patient may take from three to six spoonfuls of a solution of one drachm of citrate of iron in a pint of syrup of Tolu, and drink freely of tar water, or of a decoction of uva ursi; I also frequently prescribe turpentine and Canada balsam in the dose of six-grain pills morning and evening.

The remarkable success obtained by means of the preparations of iodine, the iodide of iron particularly, in the treatment of chronic purulent discharges, induced me to employ thsee preparations in the treatmen of gleet. The results have entirely satisfied me, and I regard the pills of the iodide of iron, preserved by the method of Gille, as the most efficacious medicament we can oppose to those chronic discharges, which are so rebellious as to cause the most experienced practitioners to despair of their cure. Four to eight pills may be taken daily; each pill contains one grain of the iodide.

Blisters to the perinæum or the inner surface of the thighs sometimes have a good revulsive effect, and occasionally cuts short the disease; sea-bathing has the same effect.

The following injections are suitable in this period of the disease: *Rose water* six ounces; *sulphate of zinc* and *acetate of lead*, each, half a drachm; or twenty or thirty grains of *tannic acid* may be substituted for the acetate of lead in the above formula. Or the patient may use an astringent injection composed of equal parts of water and Roussillon wine, with the addition of a little sugar. Here are two other prescriptions which are sometimes useful: *vinous infusion of red roses*, six ounces; *tannic acid*, one or two scruples; Mix.—*Distilled Water*, six ounces; *iron filings*, one scruple; *protoiodide of iron*, four to six grains. Mix, and take care not to shake the phial.

There are some obstinate gleets, uncomplicated with stricture, which can only be cured by the introduction of bougies. These instruments cause a mechanical irritation, an artificial gonorrhœa, which may be cured by cubebs and injections. The pewter bougies of Beniquê answer admirably for this purpose; they should be introduced gently, and left in place two or three minutes.

There is another plan which may be used as a last resource; it consists in cauterizing the whole canal of the urethra with Lallemand's caustic holder. The instrument is introduced until its extremity reaches the neck of the bladder, the canula is then drawn back and the cup of caustic is exposed and rapidly rotated as the instrument is withdrawn. There is considerable pain and scalding during micturition after the operation, but these symptoms rarely last long, and two or three cauterizations commonly put a stop to the gleet. But there are some cases which resist all the means I have described; in such, it is the surgeon's duty to recommend a tonic regimen, moderate sexual indulgence, residence in the country, mental diversion, and above all the patient should be exhorted not to think about his complaint, which is too often a cause of hypochondria and even of suicide in weak-minded individuals.

